# High prevalence of latent tuberculosis and bloodborne virus infection in a homeless population

**DOI:** 10.1136/thoraxjnl-2016-209579

**Published:** 2018-01-29

**Authors:** Robert W Aldridge, Andrew C Hayward, Sara Hemming, Susan K Yates, Gloria Ferenando, Lucia Possas, Elizabeth Garber, John M Watson, Anna Maria Geretti, Timothy Daniel McHugh, Marc Lipman, Alistair Story

**Affiliations:** 1 Centre for Public Health Data Science, Institute of Health Informatics, University College London, London, UK; 2 Department of Infectious Disease Informatics, The Farr Institute of Health Informatics Research, London, UK; 3 Institute of Epidemiology and Health Care, University College London, London, UK; 4 Royal Free London NHS Foundation Trust, London, UK; 5 Institute of Infection and Global Health, University of Liverpool, Liverpool, UK; 6 Centre for Clinical Microbiology, Division of Infection and Immunity, University College London, London, UK; 7 UCL Respiratory, Division of Medicine, University College London, London, UK; 8 Find&Treat, University College London Hospitals, London, UK

**Keywords:** clinical epidemiology, tuberculosis, respiratory infection

## Abstract

**Introduction:**

Urban homeless populations in the UK have been shown to have high rates of active tuberculosis, but less is known about the prevalence of latent tuberculosis infection (LTBI). This study aimed to estimate the prevalence of LTBI among individuals using homeless hostels in London.

**Methods:**

We performed a cross-sectional survey with outcome follow-up in homeless hostels in London. Our primary outcome was prevalence of LTBI. Recruitment for the study took place between May 2011 and June 2013. To estimate an LTBI prevalence of 10% with 95% CIs between 8% and 13%, we required 500 participants.

**Results:**

491/804 (61.1%) individuals agreed to be screened. The prevalence of LTBI was 16.5% (81/491; 95% CI 13.2 to 19.8). In UK-born individuals, a history of incarceration was associated with increased risk of LTBI (OR 3.49; 95% CI 1.10 to 11.04; P=0.018) after adjusting for age, length of time spent homeless and illicit drug use. Of the three subjects who met English treatment guidelines for LTBI at the time of the study, none engaged with services after referral for treatment. Prevalence of past hepatitis B infection was 10.4% (51/489; 95% CI 7.7 to 13.1), and 59.5% (291/489; 95% CI 55.1 to 63.9) of individuals were non-immune. Prevalence of current hepatitis C infection was 10.4% (51/489; 95% CI 7.8 to 13.1).

**Conclusions:**

This study demonstrates the high prevalence of LTBI in homeless people in London and the associated poor engagement with care. There is a large unmet need for LTBI and hepatitis C infection treatment, and hepatitis B vaccination, in this group.

**Key messages**

**What is the key question?**

- What is the prevalence of latent tuberculosis infection (LTBI) and bloodborne viral infections among homeless people in London, and what are the outcomes in those referred to healthcare services?

**What is the bottom line?**

- People experiencing homelessness in London have a very high prevalence of LTBI, hepatitis B and hepatitis C infection and coinfection, compounded by poor engagement with care.

**Why read on?**

- We report for the first time on the burden of LTBI and bloodborne viruses among homeless people in a metropolitan UK setting. The findings highlight the need to ensure recent improvements in diagnostics, and therapeutic can benefit the most vulnerable and excluded populations.

## Introduction

Homeless individuals have high rates of active pulmonary tuberculosis and often present late to healthcare services.^1^ Latent tuberculosis infection (LTBI) has been shown to be common in homeless populations in low burden countries,^2 3^ though limited data are available in the UK.

Homelessness and tuberculosis in homeless populations are both increasingly significant problems in London. Using data collected in a multiagency database about rough sleepers and the wider street population (Combined Homelessness and Information Network (CHAIN)), it is estimated that approximately 8000 people sleep rough annually in London.^4^ This number has doubled from just under 4000 in 2010, while at the same time there has been has been an annual reduction in the number of homeless hostel beds for single people and couples without dependents across England from 43 655 in 2010 to 35 727 in 2016.^5^ In 2014, it was estimated that 3.6% (89/2498) of cases with social risk factor information available had a history of tuberculosis.^6^ A study undertaken to estimate the point prevalence of active tuberculosis in London estimated that while the overall prevalence was 27 per 100 000, it was considerably greater in homeless people at 788 per 100 000.^1^

Developments in testing and treatment for LTBI and bloodborne viruses (BBVs) provide new opportunities for effective diagnosis and management.^7 8^ Despite these advances, concerns remain about LTBI treatment in this homeless population due to poor treatment adherence and the potential for severe hepatotoxicity exacerbated by high rates of alcohol-related or viral-related liver disease.^9^ It is also important to determine the current ability of health services to successfully treat those homeless people identified with a given infection before a systematic screening and treatment programme is implemented.

Due to uncertainty regarding the prevalence of LTBI and BBVs in homeless populations, doubts remain about the effectiveness and cost-effectiveness of a targeted LTBI and BBV screening strategy in this group. We therefore undertook a cross-sectional survey to estimate the prevalence of LTBI and BBVs among individuals in homeless hostels in London, a group which is broadly representative of the homeless population of the UK. We also examined outcomes of referral to healthcare services after 12 months.

## Methods

### Study population

We performed a cross-sectional survey testing for LTBI, hepatitis B, hepatitis C and HIV in residents of homeless hostels in London. The study was conducted alongside the Find and Treat (F&T) service run by the National Health Service (NHS). F&T identifies cases of active tuberculosis using digital chest radiography and supports patients to complete treatment.^10^ Recruitment for the study took place between May 2011 and June 2013, and convenience sampling was used as individuals were screened within the F&T programme. Individuals were eligible to participate in the study if they were over 18 years, resident at a homeless hostel on the day of F&T screening, had a tuberculosis screening chest radiograph by F&T (or elsewhere within the last 6 months that could be proven) and were able to provide written informed consent.

Sociodemographic and risk factor data including self-reported age, sex, history of imprisonment, history of drug and alcohol use, history of homelessness and country of birth were collected by dedicated research team using a paper-based questionnaire. The questionnaire was piloted and improved with help from homeless hostel users at the start of the study.

### Referral to NHS services

In line with the National Institute for Health and Care Excellence (NICE) guidelines, up to March 2012, individuals diagnosed with LTBI were offered advice about tuberculosis symptoms, and those coinfected with HIV were referred to local health services.^11^ After March 2012, all individuals diagnosed with LTBI who were under the age of 35 years were to be referred to local health services, reflecting new NICE guidelines for identifying and managing tuberculosis among hard-to-reach groups.^12^ Individuals with current hepatitis B or hepatitis C infection and previously undiagnosed HIV infection were referred to 14 local health services, and the outcomes were collected 12 months after referral by the research team phoning and speaking to clinicians and nurses to whom the patients were referred.

The study received approval from the East of England—Essex National Research Ethics Service Committee (no 10/H0302/5).

### Laboratory testing

Whole venous blood samples were collected to test for LTBI and BBVs. LTBI was measured using the QuantiFERON-TB Gold gamma interferon release assay (Cellestis, Australia) following the manufacturer’s instructions for interpretation (table 1).

**Table 1:** Definitions of classifications used for LTBI, hepatitis B, hepatitis C and HIV

Infection (number screened)	Classification status	Definition	Number classified (%*)
Latent tuberculosis (n=489)†	Positive‡	TB-specific antigen response >0.35 IU/mL and no evidence of active disease on clinical assessment	81 (16.5)
	Negative	TB-specific antigen response <0.35 IU/mL	408 (83.1)
Hepatitis B (n=489)†	Current	HBsAg positive, anti-HBc negative, anti-HBs negative	7 (1.4)
	Past	HBsAg negative, anti-HBc positive, anti-HBs positive (confirmed; n=43) Or HBsAg negative, anti-HBc positive, anti-HBs negative (probable past; n=8)	51 (10.4)
	Immune probably through vaccination§	HBsAg negative, anti-HBc negative¶, anti-HBs positive	140 (28.7)
	Non-immune	HBsAg, anti-HBc, anti-HBs negative	291 (59.5)
Hepatitis C (n=491)	Current	Anti-HCV positive and HCV RNA positive	51 (10.4)
	Past	Anti-HCV positive, HCV RNA negative and RIBA positive	13 (2.7)
	Uncertain history	Anti-HCV positive or equivocal, HCV RNA negative and no RIBA or insufficient sample for testing	3 (0.6)
	Negative	Anti-HCV and HCV RNA negative	424 (86.4)
HIV (n=491)	Seropositive	Anti-HIV/p24 antigen positive	5 (1.0)
	Seronegative	Anti-HIV/p24 antigen positive	486 (99.0)

*Denominator for each percentage is number screened, in first column.

†Two missing LTBI results as indeterminate and two missing hepatitis B test results due to insufficient sample for testing.

‡Further details available from Cellestis, Australia, including interpretation of controls.^34^

§Median anti-HBs level was 195 IU/L (IQR 46–945).

¶Three subjects had equivocal anti-HBc and negative anti-HBe.

HBc, hepatitis B core; HBe, hepatitis B envelope; HBs, hepatitis B surface; HBsAg, hepatitis B surface antigen; HCV, hepatitis C virus; LTBI, latent tuberculosis infection; RIBA, Recombinant ImmunoBlot Assay.

Hepatitis B surface antigen (HBsAg), core total antibody (anti-HBc) and surface antibody (anti-HBs) were detected by the Architect immunoassay (Abbott Diagnostics, Germany). Hepatitis B infection was classed as current in subjects who tested positive for HBsAg at screening with confirmation by HBsAg neutralisation. Hepatitis B was classified as confirmed past in those who were HBsAg negative, anti-HBc positive and anti-HBs positive and probable past in those who were HBsAg negative, anti-HBc positive and anti-HBs negative. For all analyses, we combine these two groups of confirmed and probable past into one group of past hepatitis B infection, and we refer to them as such throughout the rest of the paper. Non-immune hepatitis B status was defined by absence of all hepatitis B markers.

Anti-hepatitis C virus (HCV) was detected by the Vitros chemiluminescence assay (Ortho Clinical Diagnostics). Hepatitis C RNA was measured by either a real-time PCR assay based on the method described by Komurian-Pradel *et al*^13^ or the Abbott M2000 Real-Time hepatitis C assay.^14–18^ Samples reactive for anti-HCV but with undetectable hepatitis C RNA underwent anti-HCV confirmation by the Recombinant ImmunoBlot Assay (RIBA, Chiron) or the Line Immunoassay (Inno-Lia; Innogenetics). Hepatitis C infection was classed as current in anti-HCV positive subjects who tested hepatitis C RNA positive and past in those who showed undetectable hepatitis C RNA with confirmed anti-HCV positivity (table 1). HIV screening was performed by the Architect combined HIV antibody/p24 antigen chemiluminescence assay (Abbott Diagnostics).

### Analysis

The primary outcome for the study was the proportion of subjects with a positive QuantiFERON-TB Gold assay result. Based on studies in marginalised populations in the USA,^2 3 19^ we expected a minimum of 10% of participants to test positive for LTBI. To measure this within 95% CIs between 8% and 13%, we required 500 participants. Secondary outcomes were hepatitis B, hepatitis C and HIV status and outcomes in those referred to healthcare services for all infections. Data from the paper questionnaires were entered onto a Microsoft Access database created for the study. Categorisation of categorical variables, methods of assessment and treatment of missing data are presented in an online supplementary appendix. A descriptive analysis of baseline variables and their association with primary and secondary outcomes was performed. We considered age, a priori, as a confounding variable for LTBI. History of imprisonment, history of drug and alcohol use, history of homelessness and country of birth were considered as exposure variables, and a logistic regression model was used to examine the evidence for these as risk factors for LTBI. Data were analysed in STATA V.14.

10.1136/thoraxjnl-2016-209579.supp1 Supplementary file 1

## Results

### Study population

**Figure 1:**
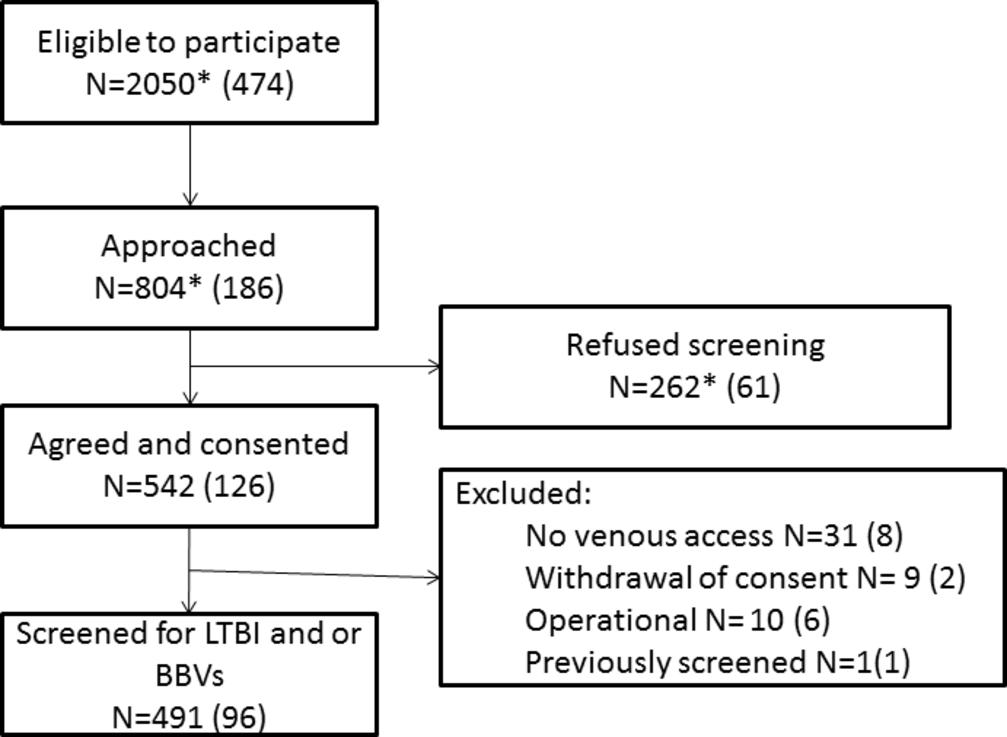
Recruitment flow chart. *It was operationally extremely intensive to collect data on the number of individuals who were eligible, approached and refused screening; therefore, these data were only collected at the start of the study. These numbers are therefore estimated on the basis of data collected at the start of study (numbers in parenthesis). BBV, bloodborne virus; LTBI, latent tuberculosis infection.

**Figure 2:**
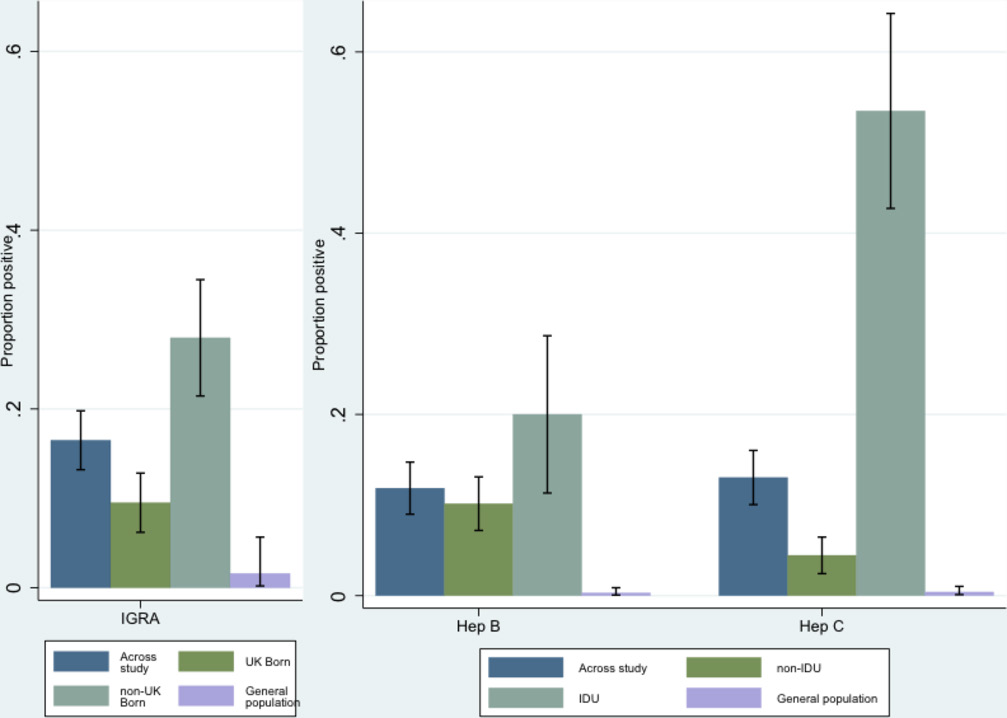
Prevalence of LTBI, hepatitis B and hepatitis C, compared with nationally representative samples. General population comparators taken from published sources: LTBI^20^; hepatitis B^35^ and hepatitis C.^21^ Hepatitis B and C results from current study were sum of current and past hepatitis B or C. Hep, hepatitis; IDU, injecting drug use; IGRA, interferon-gamma release assay; LTBI, latent tuberculosis infection.

**Figure 3:**
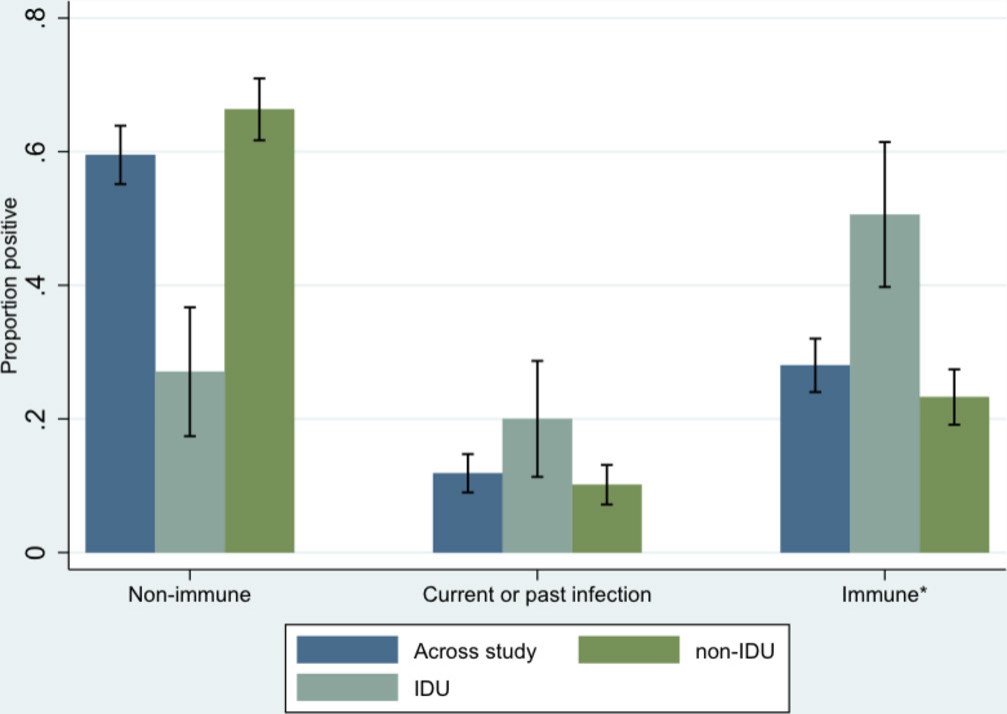
Immunity to Hep B across study and by history of IDU. *Immune due to hepatitis B vaccination. Hep, hepatitis; IDU, injecting drug use.

After accessing the F&T mobile screening service, 804 individuals were approached by research staff and invited to participate in the study. A total of 542/804 (67.4%) individuals consented to take part (figure 1). A total of 51 (9.4%) individuals were subsequently excluded, mainly due to a lack of venous access for blood sampling (n=31). A total of 491 individuals were therefore included in the analysis. A majority of participants (437/491, 89.0%) were men aged between 30 and 49 years (257/491, 52.3%), born in the UK (305/491, 62.1%) and current tobacco smokers (394/491, 80.2%). Most (443/491, 90.2%) reported to have been homeless for one or more years. Just over half (263/481, 54.7%) had spent time in prison. Drug use was common with 107/491 (21.8%) ever having smoked heroin or crack cocaine and 86/491 (17.5%) ever having injected either crack cocaine or heroin. A large number of individuals (202/477, 42.3%) had ever been concerned about their drinking or had had a health worker express concern about their alcohol consumption. Results of testing are shown in tables 1 and 2 and in figures 2 and 3.

**Table 2:** Baseline demographic and clinical characteristics for participants stratified by test results for latent tuberculosis infection and hepatitis B and C

	All	QuantiFERON-TB Gold positive		Hep B positive*		Hep C positive†	
	n	n	%	n	%	n	%
All	491	81	16.5	58	11.9	64	13.0
Age (years)							
18–29	69	8	11.6	6	8.7	3	4.3
30–49	257	39	15.2	28	10.9	43	16.7
50+	165	34	20.6	24	14.5	18	10.9
Sex							
Female	54	4	7.4	5	9.3	3	5.6
Male	437	77	17.6	53	12.1	61	14.0
Born in the UK							
Yes	305	29	9.5	29	9.5	50	16.4
No	186	52	28.0	29	15.6	14	7.5
Total time spent homeless							
<1 year	48	8	16.7	6	12.5	4	8.3
1 year	135	18	13.3	16	11.9	13	9.6
2–3 years	141	28	19.9	19	13.5	11	7.8
>3 years	167	27	16.2	17	10.2	36	21.6
Has ever spent time in prison							
No	218	35	16.1	27	12.4	12	5.5
Yes	263	45	17.1	30	11.4	50	19.0
Missing	10	1		1		2	
Illicit drug usage							
Neither	298	44	14.8	27	9.1	13	4.4
Has ever smoked heroin/crack	107	20	18.7	14	13.1	5	4.7
Has ever injected drugs	86	17	19.8	17	19.8	46	53.5
Case currently smokes cigarettes							
No	97	18	18.6	10	10.3	2	2.1
Yes	394	63	16.0	48	12.2	62	15.7
Participant or health worker ever been concerned about drinking							
No	275	51	18.5	30	10.9	24	8.7
Yes	202	28	13.9	25	12.4	36	17.8
Missing	14	2		3		4	

*Sum of current and past hepatitis B.

†Sum of current and past hepatitis C.

Note: HIV data not included to reduce risk of deductive disclosure. Hep, hepatitis.

### Latent tuberculosis infection

The overall prevalence of LTBI was estimated at 81/491 (16.5%; 95% CI 13.2 to 19.8). Prevalence was higher in those born outside of the UK (52/186, 28.0%; 95% CI 21.4 to 34.4) relative to those born in the UK (29/305, 9.5%; 95% CI 6.2 to 12.8), but both were substantially higher than the 1.6% (95% CI 0.2 to 5.7) prevalence found in patients with inflammatory bowel disease screened for LTBI before initiation of anti-tumour necrosis factor alpha therapy in the UK (figure 2).^20^ A multivariable analysis was conducted to identify risk factors for LTBI in those individuals born in the UK. There was evidence that a history of imprisonment was associated with an increased risk of LTBI (OR 3.49; 95% CI 1.10 to 11.04; P=0.018) after adjusting for age, length of time spent homeless and any illicit drug use (table 3).

**Table 3:** Logistic regression results of risk factors for latent tuberculosis infection in UK-born homeless

Risk factor	Univariable OR (95% CIs)	Multivariable OR (95% CIs)	P value*
Age			
<30	1.0	1.0	
30–49	1.36 (0.61 to 3.07)	0.69 (0.14 to 3.51)	
50+	1.98 (0.86 to 4.53)	2.04 (0.41 to 10.05)	0.07
Total time spent homeless			
<1 year	1.0	1.0	
1 year	0.77 (0.31 to 1.91)	0.32 (0.06 to 1.79)	
2–3 years	1.24 (0.52 to 2.94)	0.79 (0.18 to 3.44)	
>3 years	0.96 (0.41 to 2.29)	0.82 (0.20 to 3.32)	0.43
Has ever been to prison			
No	1.0	1.0	
Yes	1.08 (0.67 to 1.75)	3.49 (1.10 to 11.04)	0.018
Illicit drug usage			
Neither	1.0	1.0	
Has ever smoked heroin/crack	1.33 (0.74 to 2.37)	1.44 (0.49 to 4.22)	
Has ever injected drugs	1.42 (0.77 to 2.64)	2.65 (0.92 to 7.62)	0.20

*Likelihood ratio test; two indeterminate interferon gamma release assay results grouped with negative results.

### Bloodborne viruses

Current hepatitis B as confirmed by HBsAg neutralisation was 7/489 (1.4%; 95% CI 0.4 to 2.5). A large proportion of participants (51/489, 10.4%; 95% CI 7.7 to 13.1) had evidence of past hepatitis B infection. The number of individuals who were non-immune to hepatitis B was 291/489 (59.5%; 95% CI 55.1 to 63.9) and was lower for those who had ever injected drugs (23/85, 27.1%; 95% CI 17.4 to 36.7; figure 3). The majority of individuals who tested non-immune to hepatitis B (226/291, 77.7%) did not recall whether they had been previously vaccinated, and 29/291 (10.0%) reported never having received vaccination. Overall, 120 (41.2%; 120/291) had spent time in a UK prison. Only four non-immune individuals reported being vaccinated against hepatitis B more than once.

**Figure 4:**
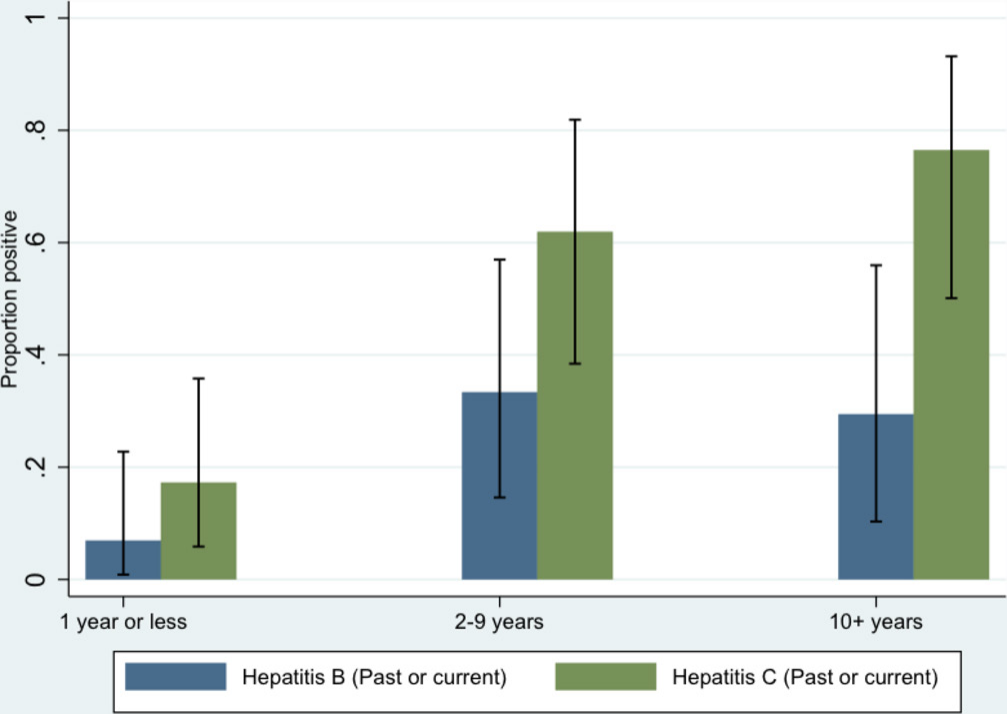
Risk of hepatitis B and C with increasing time of injecting drug use.

Among a total of 64/491 (13.0%; 95% CI 10.0 to 16.0) subjects with anti-HCV seropositivity, 51 (10.4%; 95% CI 7.8 to 13.1) tested positive for hepatitis C RNA indicating current infection. The remaining 13 subjects (2.7%; 95% CI 1.2 to 4.1) showed confirmed anti-HCV reactivity in the absence of hepatitis C RNA, indicating a resolved infection. The number of individuals with past or current hepatitis C was higher in those who had ever injected drugs (46/86, 53.4%; 95% CI 42.4 to 64.3). However, those with no injecting drug history (12/405, 3.0%; 95% CI 1.3 to 4.6) had higher levels than the general population estimates in the UK (0.4%).^21^ The highest risk of hepatitis C was found in those individuals who had been injecting drugs for more than 10 years (figure 4), but there was already an increase in prevalence when comparing injecting for 2–9 years versus 1 year or less. In those diagnosed with LTBI, the frequency of coinfection with either hepatitis B or hepatitis C (past or current) was 37.0% (95% CI 26.3 to 47.8), and coinfection with both hepatitis B and hepatitis C (past or current) was 16.2% (95% CI 9.7 to 24.7).

Prevalence of HIV seropositivity was 1.02% (95% CI 0.1 to 1.9), all cases were due to HIV-1 and all subjects were previously aware of their diagnosis.

### Clinical management and outcome

A total of 81 individuals had a positive LTBI test result, none of whom were coinfected with HIV. Three individuals who were diagnosed with LTBI after March 2012 and the introduction of updated NICE treatment guidelines were referred to local health services for chemoprophylaxis (table 4). One subject declined referral, and at 12 months follow-up, the remaining two had disengaged with services and had not started treatment.

**Table 4:** Outcomes of referral to clinical services for positive cases of latent tuberculosis, hepatitis B and hepatitis C

Outcome at 12 months	LTBI positive n (%)	HBV positive n (%)	HCV positive n (%)
Diagnosed and eligible for referral	3 (100.0)	7 (100.0)	51 (100.0)
Treatment started			
On treatment	0 (0)	0 (0)	1 (2.0)
Completed treatment	0 (0)	0 (0)	1 (2.0)
Incomplete treatment	0 (0)	0 (0)	0 (0)
Engaged with services, no treatment			
Seen, discharged, no treatment required	0 (0)	6 (85.7)	0 (0)
Under review, no treatment at present	0 (0)	0 (0)	19 (29.4)
No engagement with services			
DNA, discharged/LFU	2 (66.6)	1 (14.3)	28 (49.0)
Declined referral	1 (33.3)	0 (0)	2 (3.9)

DNA, did not attend; HBV, hepatitis B virus; HCV, hepatitis C virus; LFU, lost to follow-up; LTBI, latent tuberculosis infection.

Among subjects with a current hepatitis B infection, all seven accepted a referral; 6 of 7 were seen at least once in specialist services, none of whom was deemed to require immediate antiviral therapy over 12 months following diagnosis.

Among the subjects with current hepatitis C infection, 49/51 (96.1%) subjects accepted a referral to specialist services. Two patients initiated interferon-based treatment (3.9%; 2/51) with one having completed treatment and one still on treatment at 12 months follow-up. A further 19 (37.3%; 19/51) subjects were seen at least once over 12 months of follow-up and remained under review in the absence of treatment; 28 (54.9%; 28/51) individuals were lost to follow-up after referral.

## Discussion

This study demonstrates a burden of latent tuberculosis and BBV infections in a London homeless population at levels that are substantially higher than the general population. Although, we found that the greatest risk of LTBI was in those born outside the UK, around 10% of UK-born homeless adults were infected. UK-born individuals with a history of imprisonment had more than three times the risk of LTBI compared with other UK-born participants. During the study, referral rates for treatment for LTBI were low due to the criteria in operation at the time. Under new 2016 NICE guidelines,^22^ all those with a positive test aged up to 65 years would be referred for treatment. Therefore, instead of three people (4%; 3/81) being referred, 76 (93.8%; 76/81) would now be eligible for treatment.

Significantly higher levels of current and past hepatitis B were seen in this study compared with the general population (1.4% and 10.4%, respectively). A history of hepatitis B vaccination was higher in those reporting a history of injecting drug use, possibly as a result of targeted vaccination in this population, but there remained a substantial proportion of this homeless population who were non-immune and who would benefit from vaccination. The levels of hepatitis B are particularly important to address in this population given the risk of onward transmission due to poor living conditions and low immunisation levels. No patients were initiated on treatment; however, this is not necessarily unexpected given the prolonged clinical assessment (typically 2–3 appointments spaced out by a few months) required before treatment initiation for hepatitis B.

At 13%, the prevalence of hepatitis C infection was high. This was substantially increased in participants reporting injecting drug use, but even those without such a history had higher levels than the general population. Engagement with health services was poor in those diagnosed with current hepatitis C infection, with just over half of those referred either not attending appointments or being lost to follow-up. In only a minority of those referred was antiviral therapy initiated within 12 months. Until recently, hepatitis C care in general has been characterised by a small number of treatment initiations relative to the number of people needing and accessing care.^21^ The introduction of interferon-free regimens of short duration (typically 12 weeks) has the potential to improve engagement with care in this vulnerable population, but the impact remains to be formally investigated. In individuals diagnosed with LTBI, coinfection with either hepatitis B or C (past or current) was high at 37.0%, as was coinfection with both hepatitis B and C at 16.2%. The implications of this for LTBI treatment and risk of hepatotoxicity need to be carefully considered.

There were several strengths to our study including the sample size achieved in a population that is typically described as ‘hard-to-reach’. We managed to recruit a large number of participants as a result of long established links with homeless services (through F&T). The questionnaires used for the collection of self-reported risk factor data were developed and piloted with the target population and were improved on the basis of feedback.

Due to the nature of the population and the fact that this study was conducted alongside a busy NHS clinical service, we were not able to use a formal sampling framework for the recruitment of patients and so used convenience sampling. The requirement for individuals to be able to consent meant that our results do not include individuals who were intoxicated (by drugs or alcohol) and, therefore, is likely to under-represent those at highest risk of BBV infection.

Although it was not possible to collect data on individuals unable to consent or who were approached for screening and refused to take up the offer, the homeless population in this study included a high proportion of previous rough sleepers and people with either current or previous high-risk drug and harmful and hazardous alcohol use. Men are over-represented among homeless hostel residents, and the populations sampled are broadly demographically comparable to homeless populations nationally according to F&T data collected from extensive screening outside London, and Homeless Link’s health needs audit.^23^ We did not ask for a self-reported history of contact tracing or previous active disease as, based on our experience of the provision of health support to this population, we did not believe that these data would be reliable. The number of cases with previous active disease would be likely to be very small due to the study sample size.

We are not aware of other published data estimating the prevalence of LTBI in a large representative homeless population in the UK. Previous studies in other high-income countries (including Italy, Japan, South Korea and USA) have reported LTBI prevalence in homeless populations and found rates varying from 16% to 75.9%.^3 16–18 24^ Comparability with our findings is complicated by highly heterogeneous populations, differences between studies, including definition of homelessness used, eligibility criteria, uptake and the test used to diagnose latent tuberculosis.

A recent systematic review and meta-analysis of active tuberculosis and BBVs in homeless populations internationally found that the prevalence of hepatitis C virus infection ranged from 3.9% to 36.2% and for HIV from 0.3% to 21.1%.^15^ None of the studies testing for HIV were conducted in the UK, but one hepatitis C study, which recruited homeless individuals from shelters, special projects and medical centres in Oxford, found 26.5% of individuals positive using oral fluid testing.^25^

Our results highlight the potential value of early intervention for prevention given the increasing risk of BBVs associated with greater length of time injecting drugs. Every opportunity should therefore be taken to maximise vaccination uptake including improving healthcare interventions to those in prison. This finding is consistent with our previous work demonstrating the inverse care law with respect to influenza vaccination.^26^ Our data demonstrated that homeless people’s eligibility for influenza vaccination due to clinical risk factors was 38.9% compared with 13.0% of the general population, but only 23.7% of those eligible were vaccinated compared with national levels of 53.2%. Given this unmet need, we believe that there is a strong rationale for offering universal provision of hepatitis B vaccination to homeless people through existing services engaged with this group.^27^ Individuals who tested HBsAg positive generally maintained links with services after referral, whereas those diagnosed with hepatitis C infection showed suboptimal retention in care. Further studies are required to determine whether expanded availability of interferon-free regimens of short duration will increase engagement in this population.

Homelessness has increased dramatically in the UK since 2010, and the number of people seen rough sleeping has doubled nationally.^28 29^ These populations represent the extreme end of health inequalities in high-income countries and experience a high burden of preventable morbidity and mortality from infectious and non-infectious diseases.^30 31^ Our study demonstrates for the first time the high prevalence of undiagnosed LTBI and hepatitis B and C, in homeless populations in the UK and a large unmet need for hepatitis B vaccination. Our findings also clearly illustrate the requirement for intensive case management and ongoing support to ensure that testing can translate into treatment opportunities. The very high rates of coinfection demonstrated highlight the importance of service integration through combined testing and treatment pathways.^32^ NICE now recommends that persons accessing targeted mobile radiology should be offered tests for BBV,^22^ and our data provide the basis to estimate the cost-effectiveness of this approach. The recent national collaborative TB strategy^33^ commits to new investment in a national outreach service in line with the proven F&T outreach model.^10^ Our findings reinforce the need for an integrated screening and treatment support model, while highlighting the ongoing complexity found in this population plus the support they will require through such services.
