# Supplementary material for: High prevalence of latent tuberculosis and bloodborne virus infection in a homeless population
Source: Thorax. 2018 Jan 29;73(6):557–64. doi: 10.1136/thoraxjnl-2016-209579 (PMC5969342; doi:10.1136/thoraxjnl-2016-209579)
Supplement: Supplementary file 1 [file thoraxjnl-2016-209579supp001.pdf]

Table 1. Data sources of variables, methods of assessment, missing data rules and details of subgroups.

Variable	Methods of assessment	Units	For multivariable analysis, missing values classified as:	Subgroups chosen for analysis
Age [years]	Self-report	Years	NA	18-29;<br>30-49;<br>50+
Sex	Self-report	NA	NA	Male;<br>Female
Born in the UK	Self-report	NA	NA	Yes;<br>No
Total time spent homeless	Self-report	Years	NA	<1year;<br>1 year;<br>2-3 years;<br>>3 years
Ever spent time in prison	Self-report	NA	No	No;<br>Yes;<br>Missing
Illicit drug usage	Self-report	NA	NA	None;<br>Ever smoked heroin / crack;<br>Ever injected drugs<br><br>Highest risk group for BBVs chosen i.e. if smokes and injects then classified as injects.
Case currently smokes cigarettes	Self-report	NA	NA	Yes;<br>No
Participant or health worker ever been concerned about drinking	Self-report	NA	No	Yes;<br>No
